# Specificity Assessment of CRISPR Genome Editing of Oncogenic EGFR Point Mutation with Single-Base Differences

**DOI:** 10.3390/molecules25010052

**Published:** 2019-12-22

**Authors:** Taegeun Bae, Hanseop Kim, Jeong Hee Kim, Yong Jun Kim, Seung Hwan Lee, Byung-Joo Ham, Junho K. Hur

**Affiliations:** 1 Department of Medicine, Graduate School, Kyung Hee University, Seoul 02447, Korea; btg417@naver.com; 2 National Primate Research Center, Korea Research Institute of Bioscience and Biotechnology, Cheongju 28116, Korea; contac113@kribb.re.kr; 3 Department of Biomedical Science, Graduate School, Kyung Hee University, Seoul 02447, Korea; jeonghee3698@naver.com (J.H.K.); yjkim1@khu.ac.kr (Y.J.K.); 4 Department of Pathology, College of Medicine, Kyung Hee University, Seoul 02447, Korea; 5 Department of Psychiatry, Korea University Anam Hospital, Korea University College of Medicine, Seoul 02841, Korea; 6 Department of Biomedical Sciences, Korea University College of Medicine, Seoul 02841, Korea; 7 Brain Convergence Research Center, Korea University Anam Hospital, Seoul 02841, Korea

**Keywords:** CRISPR, off-target, specificity, PAM, single-base precision

## Abstract

In CRISPR genome editing, CRISPR proteins form ribonucleoprotein complexes with guide RNAs to bind and cleave the target DNAs with complete sequence complementarity. CRISPR genome editing has a high potential for use in precision gene therapy for various diseases, including cancer and genetic disorders, which are caused by DNA mutations within the genome. However, several studies have shown that targeting the DNA via sequence complementarity is imperfect and subject to unintended genome editing of other genomic loci with similar sequences. These off-target problems pose critical safety issues in the therapeutic applications of CRISPR technology, with particular concerns in terms of the genome editing of pathogenic point mutations, where non-mutant alleles can become an off-target with only a one-base difference. In this study, we sought to assess a novel CRISPR genome editing technique that has been proposed to achieve a high specificity by positioning the mismatches within the protospacer adjacent motif (PAM) sequence. To this end, we compared the genome editing specificities of the PAM-based and conventional methods on an oncogenic single-base mutation in the endothelial growth factor receptor (EGFR). The results indicated that the PAM-based method provided a significantly increased genome editing specificity for pathogenic mutant alleles with single-base precision.

## 1. Introduction

The CRISPR system is a microbial immune system found in bacteria and archaea [1]. Among the CRISPR systems, the class II subtypes, composed of single-unit proteins such as CRISPR-Cas9 have been reported to effectively and easily control target genes in mammalian systems [2,3]. The type II CRISPR-Cas9 genome editing system is composed of protein effector Cas9 which is directly involved in DNA cleavage and guide RNA, which has a complementary sequence to the target DNA [2,4,5]. Based on in-silico prediction, effective guide RNAs with low off-target cleavage can be selected, constructed, and applied to in vivo systems after testing in the various pre-clinical system [6]. CRISPR has off-target issues, where non-target genomic loci with similar sequences to the target site are at risk of undergoing genome editing, which could result in unpredictable side effects. This is particularly true in the application of CRISPR for therapeutic purposes, wherein the off-target effect raises serious safety concerns.

Several studies have used CRISPR-based gene therapy to perform genome editing on mutations associated with diseases including cancer [2,7,8,9]. Oncogenic mutants usually involve gain-of-function changes in the DNA sequence in cancer cells, where an unmutated normal allele may still remain. To perform CRISPR gene therapy with precision, the mutated oncogenic allele should be selected for genome editing, such that the normal allele with a one-base difference is effectively distinguished. As several oncogenes that are associated with cancerous cell phenotypes are known, if oncogenic mutations are detected by medical examination, it is possible to predict the prognosis of the cancer cells when these carcinogenic mutations are removed using CRIPSR genome engineering tools.

For implementing precise genome editing of oncogenic mutations, the guide RNA sequences are important factors for determining the specificity. From a molecular point of view, two elements are necessary for DNA target selection by CRISPR. The first is the sequence complementarity to the guide RNAs, and the second is the presence of a protospacer adjacent motif (PAM) sequence, which is “NGG” for *Streptococcus pyogenes* Cas9. Generally, for genome editing, CRISPR is designed to include the mutation in the guide RNA sequence. However, recent studies have shown that CRISPR is sensitive to the requirement of the PAM (NGG) sequence, which is essential for CRISPR-Cas9 to recognize and cut the target DNA. Based on this information, several studies have implemented the design of guide RNA to position a PAM sequence at the targeted mutation [10,11]. In a recent study, CRISPR-Cas9 was applied to cancer cells caused using *KRAS* mutations, which resulted in the repression of the proliferation of cancer cells and a gradual decrease of the tumor burden caused by cancer cells [11].

Among the oncogenic mutations that can be subjected to CRISPR gene therapy are point mutations in EGFR, which are often found in lung cancer and are of high interest. EGFR is a group of membrane receptors that regulate cell growth, division, survival, and death. Many carcinomas show increased expression of EGFR in tumor tissue cells [12]. In particular, a large number of mutated genotypes (e.g., exon 19 deletion, exon 21 missense substitution) of activated EGFR have been reported in lung cancer patients, and tumor tissues with increased mutations are known to be more invasive and metastatic than normal cells [13]. Conventional chemotherapy (e.g., radiation and chemotherapy) has been applied, but the prognosis of patients is usually poor [14]. Tyrosine kinase inhibitors (gefitinib and erlotinib), which are currently used as first-line treatments by targeting EGFR, also have the drawback of inducing secondary mutations and tend to be resistant to further treatment [15]. For this reason, there is a need for personalized medical technology that can directly target various EGFR mutation genes reported in lung cancer patients. As one of these techniques, CRISPR genome engineering technology, which can selectively remove the EGFR mutation in cancer cells and suppress tumor progression, has been increasingly attracting attention [16,17,18,19].

In this study, we sought to assess the specificity of the PAM-based guide RNA design in comparison to the conventional guide RNA design, where the mutations are positioned within the guide RNA sequence. To this end, we showed how guide RNA design can be used to increase the specificity of CRISPR genome editing on an oncogenic EGFR point mutation (2573T > G, L858R) without affecting normal EGFR genes that only differ by one base. Next, we analyzed the genome-wide off-target effect of the PAM-based CRISPR method for the EGFR 2573T > G mutation and found no detectable insertions or deletions at the potential off-target sites. Together, our data suggest that the design of guide RNA based on information on PAM provides an increased specificity to the CRISPR genome editing of pathogenic mutations with single-base changes.

## 2. Results

### 2.1. Tested Guide RNA Designs for Targeting Oncogenic Point Mutation in EGFR (2573T > G)

**Figure 1:**
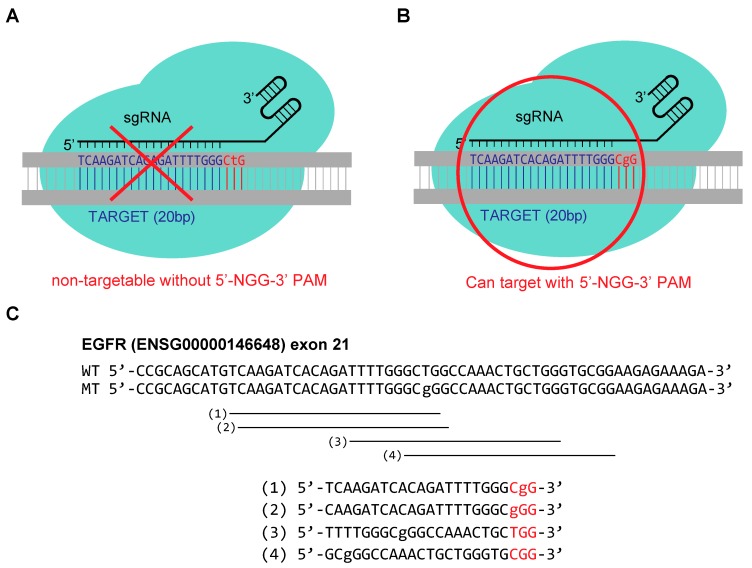
Schematic of the protospacer adjacent motif (PAM)-based guide RNA design for the targeting of the 2573T > G endothelial growth factor receptor (EGFR) point mutation. (**A**) Shown are the sequence of wild type EGFR (2573T), in grey, and the ribonucleoprotein complex comprised of CRISPR-Cas9 and guide RNA, in cyan. The PAM sequence is shown in red characters. (**B**) Schematic of the mutant sequence of EGFR (2573T > G), CRISPR-Cas9, and guide RNA. Lower-case letters indicate the 2573T > G mutation of EGFR. (**C**) Design of the four sgRNAs with single-base mismatches in PAM or within the guide RNA complementary sequences, indicated in lower-case letters.

Guide RNAs were designed to allow CRISPR-Cas9 based sequence-specific targeting to effectively distinguish the normal wild type (WT) allele of EGFR (2573T) (Figure 1A) and conduct genome editing on the mutated sequence (MT) of EGFR (2573T > G) (Figure 1B). For the PAM-based guide RNA design, the thymidine at position 2573 (2573T) of the WT EGFR allele was positioned within the PAM sequence as’ 5′-NTG-3′. The “NTG” PAM sequence configuration effectively excluded the WT allele from targeting by CRISPR-Cas9 (Figure 1A). On the other hand, the guanosine at position 2573 (2573T > G) of the mutant EGFR allele, which comprises the 5′-NGG-3′ PAM sequence, was readily targeted by CRISPR-Cas9 (Figure 1B).

We sought to assess the specificity of a PAM-based guide RNA design, as well as compare the specificity with other guide RNAs designed with base mismatches within the guide RNA sequences (Figure 1C). To this end, three other guide RNAs were also designed to position mismatches caused by the 2573T > G mutation within the guide RNA sequences The four guide RNAs were designed as single guide RNAs (sgRNAs) with the following characteristics: (i) sgRNA matches the mutated sequence at first G of 5′-NGG-3′ PAM sequence (PAM-based design, sgRNA-1); (ii) sgRNA matches the mutated sequence at N of 5′-NGG-3′ PAM sequence (sgRNA-2); (iii) sgRNA matches the mutated sequence at sgRNA position 9, which is 12 nucleotides away from PAM, sgRNA-3; (iv) sgRNA matches the mutated sequence at sgRNA position 3 (18 nucleotides away from PAM, sgRNA-4). The mismatch positions of sgRNA-3 and sgRNA-4 represent the “seed region” and “PAM distal region” of the CRISPR-Cas9 target sequence, respectively.

### 2.2. Constructing a Dual Fluorescence Reporter System for the Quantification of Genome Editing Efficiency

**Figure 2:**
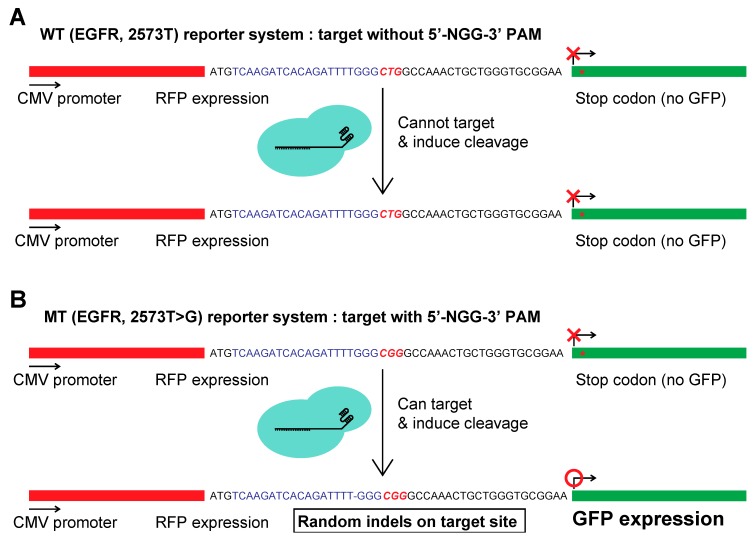
Schematic of the targeting of the RFP-GFP reporter system by CRISPR-Cas9. (**A**,**B**) Shown are the reporter construct with the wild type EGFR (2573T) and mutant EGFR (2573T > G) sequence flanked by red fluorescent protein (RFP) and green fluorescent protein (GFP). The PAM-based sgRNA sequence and its PAM are shown in blue and red, respectively.

Next, we sought to construct a quantitative reporter system to assess the specificities of the sgRNAs in the CRISPR genome editing system. To this end, we used two dual fluorescence CRISPR reporter systems [20], inserting the WT and mutant DNA sequences of EGFR exon 21 to construct the WT and MT reporters (Figure 2A,B). In the dual fluorescence CRISPR reporter systems, both the WT and MT reporter system were found to normally express only red fluorescence protein (RFP) under the constitutively active cytomegalovirus (CMV) promoter, as the expression of green fluorescence protein (GFP) is blocked by stop codons present between RFP and GFP. Upon genome editing with the CRISPR system, the stop codons were removed, and the reporter started to express both RFP and GFP. Hence, the system allows for the dual fluorescence-based quantification of the efficiency of the CRISPR genome editing system. As for the four sgRNAs used to target the EGFR mutant, if the guide RNAs was perfectly specific to the mutant allele, the designed CRISPR-Cas9s systems should be unable to conduct genome editing on the WT reporter, and thus only edit RFP, such that GFP would not be expressed (Figure 2A). On the other hand, the designed sgRNAs were expected to induce CRISPR-Cas9 genome editing on the MT reporter to cleave and induce insertions and deletions (indels) at the target site, such that GFP would be expressed by frameshift (Figure 2B). Therefore, we anticipated that the MT and WT reporters would provide quantitative information on the genome editing efficiencies and off-target effects of the sgRNAs.

**Figure 3:**
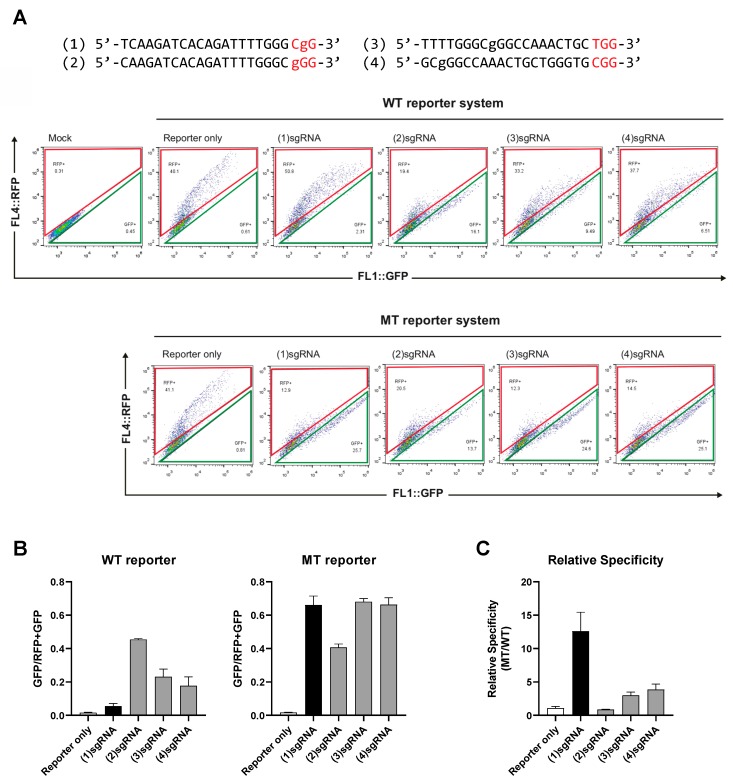
Comparison of the specificities of the PAM-based and sequence-complementarity methods for genome editing of targets with single-base differences. (**A**) Flow cytometry analyses of the genome editing specificities of the sgRNAs targeting the RFP-GFP dual reporters; (**B**) the graphs represent the ratio of the GFP population divided by whole population (RFP+GFP) in the WT and MT reporter systems, respectively. (**C**) The relative specificities are calculated by dividing the ratio of the green fluorescence protein (GFP) population in the MT reporter by that of the WT reporter. Lower-case letters indicate the mutated sequence (2573T > G). Red letters indicate the PAM sequences.

Subsequently, we sought to assay the genome editing efficiency of the sgRNAs by using the dual fluorescence reporter systems. To this end, we transfected the reporters and CRISPR-Cas9 into cells and then performed flow cytometry using the RFP and GFP signals of the reporter (Figure 3A, Supplementary Figure S1A,B). First, we tested the reporter system by transfecting only the reporters. Analyses of the dual fluorescence showed that, for both the MT or MT reporters, the RFP-only cell counts were above 30% of the total cell population, while the RFP + GFP double-positive cell counts were only about 0.5%. This result suggests that the reporter signals were robust and could provide a wide dynamic range. Next, to assess the off-target effect of the sgRNAs on the WT allele, we co-transfected the WT reporter with the four sgRNA and CRISPR-Cas9 (Figure 3A,B). The analyses and quantifications of the fluorescence signal showed that CRISPR-Cas9 mediated genome editing by PAM-based sgRNA-1 resulted in only subtle changes in the RFP-GFP profile. On the contrary, the other three sgRNAs induced notable shifts in the fluorescence profile towards the RFP-GFP double-positive signals. Interestingly, sgRNA-2 showed the highest rate of off-target issues, which is consistent with the notion that a base mismatch at the “N” position of PAM is well-tolerated by CRISPR-Cas9. Together, these results suggest that the off-target effect of sgRNA-1 on the WT sequence is significantly lower than that of sgRNA-2,3,4.

We then assayed the genome editing efficiency of the sgRNAs on the MT reporter, which is the actual on-target for genome editing (Figure 3A,B). As anticipated, all four sgRNAs showed notable shifts of the dual fluorescence reporter toward RFP + GFP double-positive signals, indicating that the sgRNAs were effective genome editing systems. Next, we sought to assess the specificities of the sgRNAs in distinguishing the one-base differences of WT and MT sequences. Comparing the target specificities (Figure 3C), we found that sgRNA-1, with the mismatch in the first “G” position of the “NGG” PAM sequence, significantly outperformed the other sgRNAs in terms of the selective genome editing of the mutant allele.

### 2.3. Analyses of the Indel Patterns and Genome-Wide Off-Targets of the PAM-Based Guide RNA

**Figure 4:**
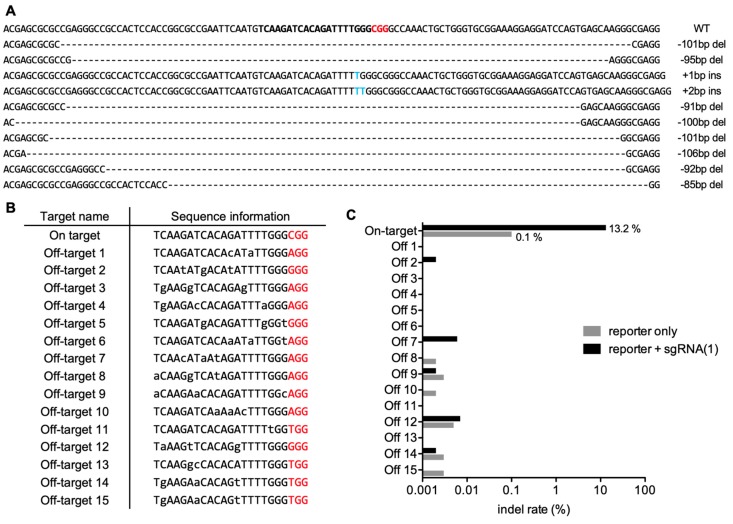
Indel patterns and off-target analyses by T7E1 assay. (**A**) Shown are the most abundant 10 indel patterns of the on-target site by genome editing with sgRNA-1. The PAM sequence and insertions are indicated by red and blue characters, respectively. (**B**) Shown are the sequences of potential 15 off-target sites. Mismatches to the on-target sequence and PAM sequences are indicated by lower-case and red characters, respectively. (**C**) Shown are the indel rates of the on-target and the 15 candidate off-target sites that were quantified by targeted deep sequencing.

As the sgRNA-1 showed a highly selective genome editing of the EGFP point mutation, we further analyzed this sgRNA with respect to its indel patterns and potential genome-wide off-targets (Figure 4A–C, and Supplementary Figure S2). Genome editing by PAM-based sgRNA-1 resulted in notable indel rates (13.2%) with various indel patterns (Figure 4A–C) within the general repertoire of the indel patterns found in CRISPR-Cas9 genome editing. On the contrary, the indel rate of the on-target site without genome editing by sgRNA-1 was only 0.1%. As indel rates below 0.1% could be caused by PCR effort and thus considered insignificant, together the results suggested that sgRNA-1 induced significant genome editing at the on-target site.

We next assayed whether the PAM-based sgRNA-1 could induce unexpected off-target effects in other genomic loci with similar sequences. To this end, we first conducted a human genome-wide computational search and found 15 potential off-targets with near-perfect sequence matches to the sgRNA-1 (Figure 4B). We then transfected the sgRNA-1 and CRISPR-Cas9, along with the MT or WT reporters, into the cells and extracted the genomic DNA after two days. Finally, we assessed the off-target effect via targeted deep sequencing (Figure 4C and Table 1) and T7E1 assay (Supplementary Figure S2). We observed that the indel rates of all off-target sites were below 0.1 %. The results showed that sgRNA-1 based CRISPR genome editing did not induce significant off-target effects in any of the 15 computationally-predicted off-target sites.

## 3. Discussion

In this study, we characterized the specificity of single-base precision of the CRISPR method based on the design of guide RNA based on PAM sequence information [19,21,22]. We show that the PAM-based method increases the specificity of CRISPR genome editing towards a well-known point mutation in the EGFR gene that is often found in lung cancer patients. Generally, the targeting of CRISPR base editing is implemented to ensure that the target locus selection depends on the base complementarities between the guide RNA and the DNA target sequences. However, we showed that target specificity via sequence complementarity of guide RNA and target DNA is insufficient to achieve the level of single-base precision that is required for certain mutations such as the oncogenic 2573T > G EGFR mutation, which is associated with lung cancer. The number and position of mismatches between the guide RNA and the target DNA are important factors that determine the specificities of the allele discrimination, and also the possibility of the off-target problems [23]. We also noted that the PAM-based method did not result in a significant increase in off-target problems.

To assess the precision of PAM-based CRISPR genome editing, we assayed the specificity of a PAM-information based method that circumvents the CRISPR target selection mechanism of RNA-DNA complementarity. To this end, we designed the guide RNAs so that the single-base mutations were placed not only within the RNA/DNA hybrid but also within the PAM sequence, which recognized the CRISPR-Cas9 protein itself. In the study, we compared the specificities of guide RNAs and found that sgRNA designed for PAM-dependent target selection outperformed the conventional CRISPR targeting, the latter of which depends entirely on the matching of the sgRNA sequence to the target DNA. The enhanced specificity of the PAM-based method can be beneficial for developing therapies for diseases, as the decreased off-target problems would lower the danger of unanticipated side-effects.

Another advantage of the single-base precision CRISPR method shown in this study is its simplicity and flexibility for use in medical applications to intervene in pathogenic single-base mutations. The CRISPR technique can be applied to lung cancer patients, as well as to normal people, with specific gene mutations that can be used to prevent carcinogenesis in advance. Our work demonstrated the concept of an enhanced CRISPR method applicable for the elimination of various oncogenic genes found in lung cancer, as well as other types of cancer, thus showing the potential of personalized treatment to individual patients in the future. Furthermore, the PAM-based CRISPR methods could be applied not only to address point mutations but also to conduct gene correction by providing an adequate DNA template.

Furthermore, while this study utilized a CRISPR-Cas9 where the PAM is determined as “NGG,” several other CRISPR systems exist that contain different PAM sequences with high sensitivity for base mismatches [24]. Therefore, it would be possible to apply different types of CRISPR systems with a diverse PAM repertoire, such as “TTN” PAM of CRISPR-Cas12a (Cpf1), for tailored matching to the various pathogenic single-base mutations. On average, human genomes contain 4 to 5 million single-nucleotide polymorphisms (SNPs), or approximately one SNP in every 1000 bases. In addition, the prevalent C-to-T conversion by methyl-cytosine deamination induces further somatic single-base mutations, among which certain pathogenic mutations could be targeted by CRISPR genome editing. In conclusion, we anticipate that the PAM sequence-based guide RNA design method would contribute to the application of CRISPR genome editing in the development of personalized therapeutic treatments with higher specificities and lower off-target effects.

## 4. Materials and Methods

### 4.1. Cell Culture and Transfection

A549 cells were cultured in DMEM with 10% fetal bovine serum (FBS) and 1% antibiotics.

The cells (1 × 10^5^) were seeded into 24-well plates and transfected with the reporter vector (300 ng), sgRNA plasmid (300 ng), and SpCas9 plasmid (400 ng) using Lipofectamine 2000 (Invitrogen, Waltham, MA, USA). The transfected cells were cultured for 48 h and sorted by FACS using RFP and GFP. After FACS analysis, the genomic DNA was isolated from the cells using the DNeasy Blood & Tissue Kit (Qiagen, Venlo, Netherlands) for subsequent analyses.

### 4.2. Targeted Deep Sequencing

The on-target sites for the reporter system were amplified by PCR using Phusion High-Fidelity DNA Polymerase (Thermo Fisher Scientific, Waltham, MA, USA). The PCR amplicons were subjected to paired-end read sequencing using an Illumina iSeq 100 instrument. The sequencing data were analyzed using the Cas-Analyzer. The frequencies of the indels (Insertions and deletions) located 3bp upstream of the PAM were considered to be the mutations induced by CRISPR-Cas9.

### 4.3. T7E1 Assay

PCR amplicons were obtained for the predicted off-target candidates from genomic DNA extracted after the treatment of cells with CRISPR-Cas9 for 48 h. For each amplicon, the denaturation and re-annealing process were carried out using a PCR machine, followed by the DNA cleavage reaction with the T7E1 enzyme at 37 °C for 25 min. The reaction was terminated by adding a stop buffer (100 mM EDTA, 1.2% SDS. The cleaved DNA fragment was separated by 2% agarose gel electrophoresis.

## Figures and Tables

**Table 1:** DNA Primer Sequence Information for deep sequencing assay of off-target sites. Mis-matched nucleotides are shown in lower-case letters.

Target Gene (Primer Direction)	Primer Sequence (5′ to 3′)	Off-Target Sequence
EGFR_Off_1_F1	TAAATTGAGTGCAGAGCCTTG	TCAAGATCACAcATaTTGGGAGG
EGFR_Off_1_R1	GCAATGGTACCCTTTTCTTC	
EGFR_Off_1_F2	AAACCCTGTGTTTGGAATTCA	
EGFR_Off_1_R2	TTTGTTGTCCTTATATGTCATTGTG	
EGFR_Off_2_F1	TCAGAGCCCAGTTTATCATAG	TCAAtATgACAtATTTTGGGGGG
EGFR_Off_2_R1	GTAAATTAGCTGGTCTCAGG	
EGFR_Off_2_F2	GGCATCAAGTAAATTACTCAAAACA	
EGFR_Off_2_R2	CATGTAGCCAAAACAAAACAAACA	
EGFR_Off_3_F1	ATTCAACAACGATCTGTTGTG	TgAAGgTCACAGAgTTTGGGAGG
EGFR_Off_3_R1	AAAGCAAAACCCCAAAGCGA	
EGFR_Off_3_F2	TCTAGTGGAGACAGATGTTAAAC	
EGFR_Off_3_R2	GGGCTTTCCTCTCTTCTGT	
EGFR_Off_4_F1	TGCATTTTGACTCTTCTCGA	TgAAGAcCACAGATTTaGGGAGG
EGFR_Off_4_R1	AGAAGAAAGCTTCTGATGCC	
EGFR_Off_4_F2	ATATCCAATAACAACAATACTAGC	
EGFR_Off_4_R2	CCATCATTGAATTGAGGTCAC	
EGFR_Off_5_F1	GGAAATCAAATTTGGGGTTG	TCAAGATgACAGATTTgGGtGGG
EGFR_Off_5_R1	CTGCTGTTCCCCAACTTATT	
EGFR_Off_5_F2	TCTGAGACTGGGTCATTCAT	
EGFR_Off_5_R2	GCTTTTTACAACTATCTTACTAATAAC	
EGFR_Off_6_F1	CCTTGTTGATTTACATTGATGTG	TCAAGATCACAaATaTTGGtAGG
EGFR_Off_6_R1	CATTCCATAACATTCTCAGGT	
EGFR_Off_6_F2	CCAATGTAAATCAACAAGGGT	
EGFR_Off_6_R2	AATCATGAACATATTTATGCTTTCC	
EGFR_Off_7_F1	CAAACTCACAATTGTGAGGG	TCAAcATaAtAGATTTTGGGAGG
EGFR_Off_7_R1	AGCAGAGCATGGAGCTCATA	
EGFR_Off_7_F2	ATGGACCGGGTGGCTTAAA	
EGFR_Off_7_R2	AATGAAGCATGGTGACAGAC	
EGFR_Off_8_F1	ACCAGGATGGTCTTGATCTC	aCAAGgTCAtAGATTTTGGGAGG
EGFR_Off_8_R1	AAGTGTTTAAAACAGTGCCA	
EGFR_Off_8_F2	CTGCCCAGGCTTCATCTTAA	
EGFR_Off_8_R2	CCAGGCTGAAATGATCAAAG	
EGFR_Off_9_F1	ATTTCAAAGGGTGGGGCTTT	aCAAGAaCACAGATTTTGGcAGG
EGFR_Off_9_R1	GAAGTCTCAGATCAAGGTCC	
EGFR_Off_9_F2	TTGGGACTTGTCATCCTTTT	
EGFR_Off_9_R2	GGAGAAGAGCATGAGTGCTA	
EGFR_Off_10_F1	TAGACTAGTCACCAGAATTCC	TCAAGATCAaAaAcTTTGGGAGG
EGFR_Off_10_R1	TCACCATGCAGTTGTACATA	
EGFR_Off_10_F2	CAAGGAAGACAAAAGAGACA	
EGFR_Off_10_R2	TCCCCAGTCTGTTCTCTCTT	
EGFR_Off_11_F1	GTTATGTGAGGTGTTTGTGT	TCAAGATCACAGATTTTtGGTGG
EGFR_Off_11_R1	AGAACACACCATGTTAGAGG	
EGFR_Off_11_F2	TGATGATAATTTCATGTGTGTTACC	
EGFR_Off_11_R2	ACTCTCTTTGGTGAGAAGGA	
EGFR_Off_12_F1	TACAGTTGTGTGGCTTTCGA	TaAAGtTCACAGgTTTTGGGGGG
EGFR_Off_12_R1	GCCGTCTCAATACTTGTGAA	
EGFR_Off_12_F2	AACAACTATGGTATGGGCCA	
EGFR_Off_12_R2	GGTAAAACCCCATCTACTAAAAA	
EGFR_Off_13_F1	GCCTCTTATCTGAACGAGAA	TCAAGgcCACAcATTTTGGGTGG
EGFR_Off_13_R1	ATTAGTTGCAGTTCAAAGCC	
EGFR_Off_13_F2	AAATCTACTTGGAGCAATGC	
EGFR_Off_13_R2	TTTTTGGTAGGAGCCTGCAG	
EGFR_Off_14_F1	AGTGTTTCGATAGATGGAGG	TgAAGAaCACAGtTTTTGGGTGG
EGFR_Off_14_R1	CTACCCAAAACCTTTGTCCC	
EGFR_Off_14_F2	AGAAGAGCACGAGTGGTAAA	
EGFR_Off_14_R2	CACCCTTTTTCTTCCTCCAT	
EGFR_Off_15_F1	GGTGGGGAAAAAAGTTTTTGG	TgAAGAaCACAGtTTTTGGGTGG
EGFR_Off_15_R1	CTACACCTTCTTTTCCCGAC	
EGFR_Off_15_F2	GAGTGACGAGGAGGAGGAAA	
EGFR_Off_15_R2	TCAACACCCTTTTCCCCAT	
EGFR_on-target_F	TACTTGAAGCTGTCCTTCCC	TCAAGATCACAGATTTTGGG
EGFR_on-target_R	CCGTCGTCCTTGAAGAAGAT	

## Supplementary Materials

The following are available online, Figure S1: Flow cytometry analyses of the genome editing specificities of the sgRNAs targeting the RFP-GFP dual reporters. (A) and (B) indicate the fluorescence profiles of biological replicates, Figure S2. T7E1 analyses of the 15 candidate off-target sites. (A) Off-target analyses of off-targets using MT reporter and sgRNA(1). (B) Analyses of off-targets using WT reporter and sgRNA(1).
