# Supplementary material for: Specificity Assessment of CRISPR Genome Editing of Oncogenic EGFR Point Mutation with Single-Base Differences
Source: Molecules. 2019 Dec 22;25(1):52. doi: 10.3390/molecules25010052 (PMC6982904; doi:10.3390/molecules25010052)
Supplement: Supplementary file 1 [file molecules-25-00052-s001.zip › molecules-644667-supplementary.pptx]

## Slide 1
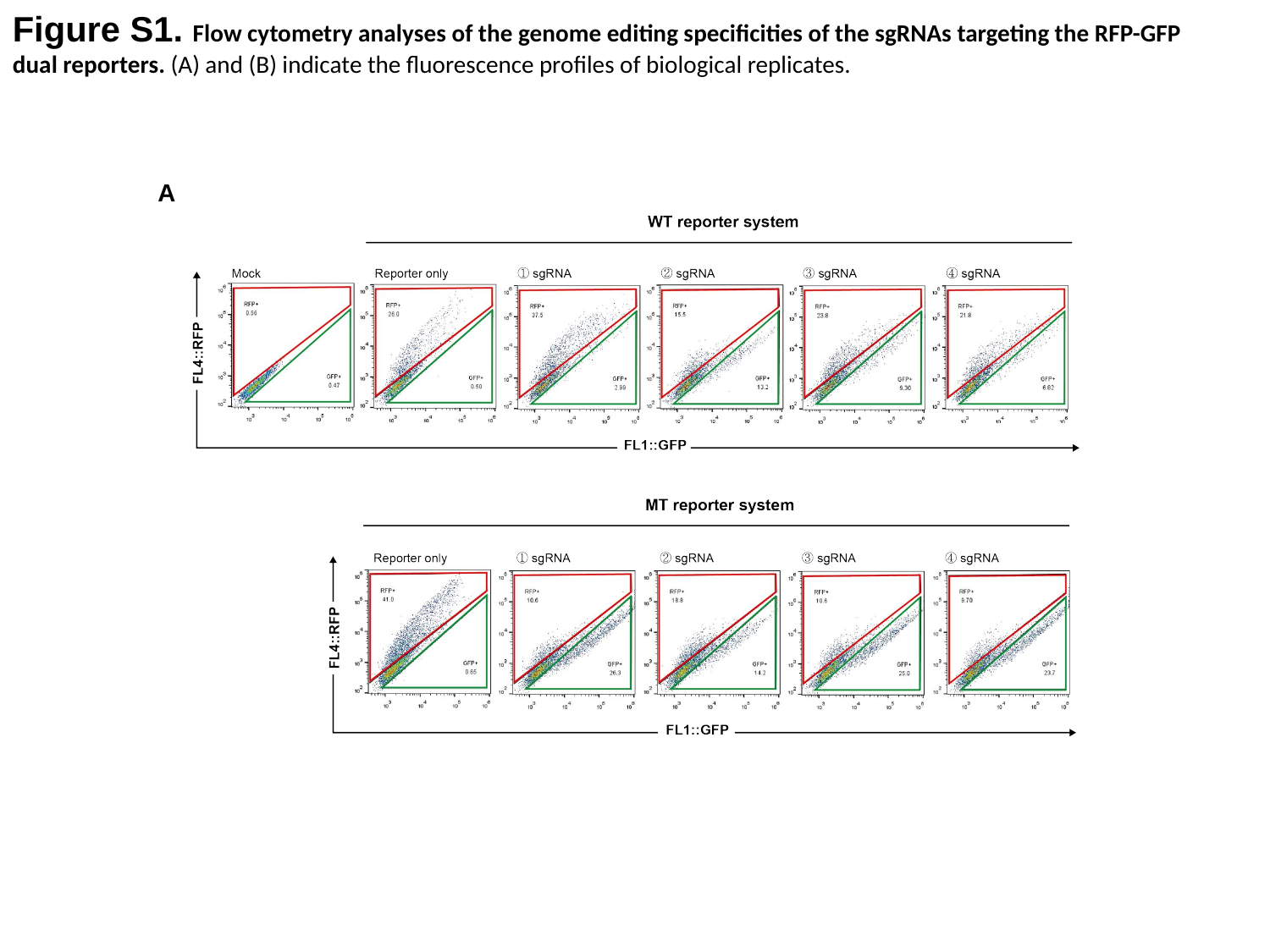

Figure S1. Flow cytometry analyses of the genome editing specificities of the sgRNAs targeting the RFP-GFP dual reporters. (A) and (B) indicate the fluorescence profiles of biological replicates.
A

## Slide 2
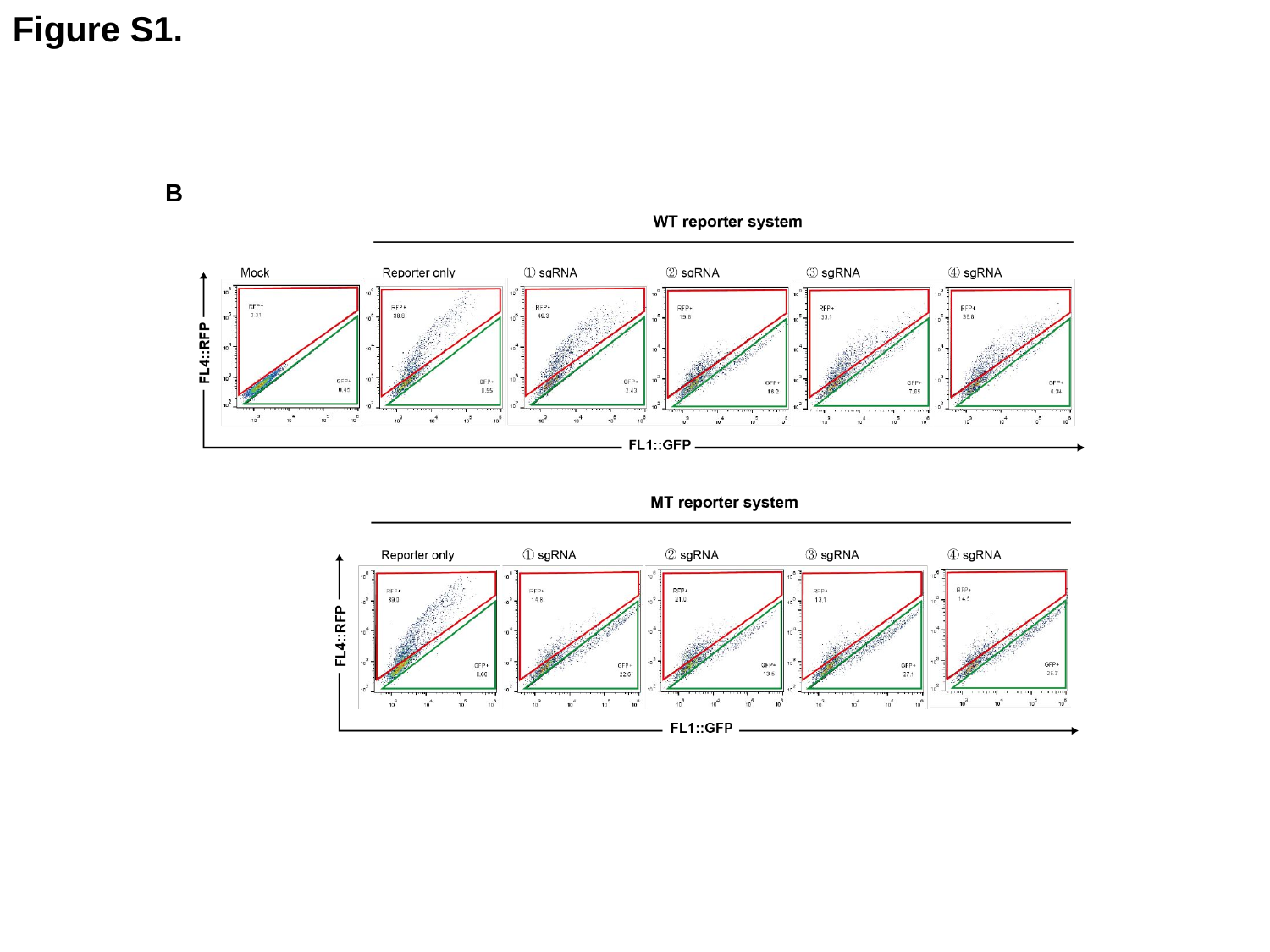

Figure S1.
B

## Slide 3
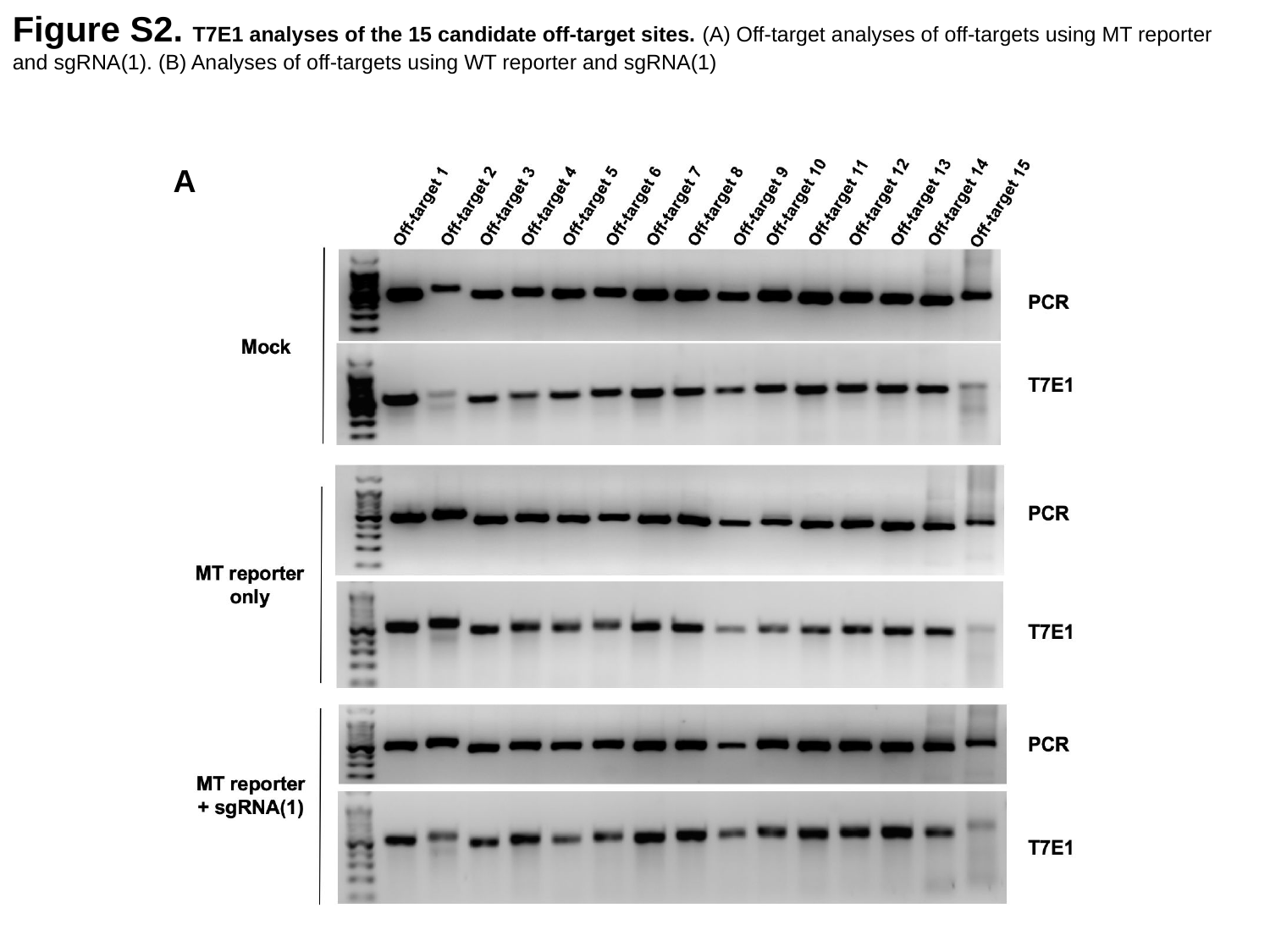

Figure S2. T7E1 analyses of the 15 candidate off-target sites. (A) Off-target analyses of off-targets using MT reporter and sgRNA(1). (B) Analyses of off-targets using WT reporter and sgRNA(1)
A

## Slide 4
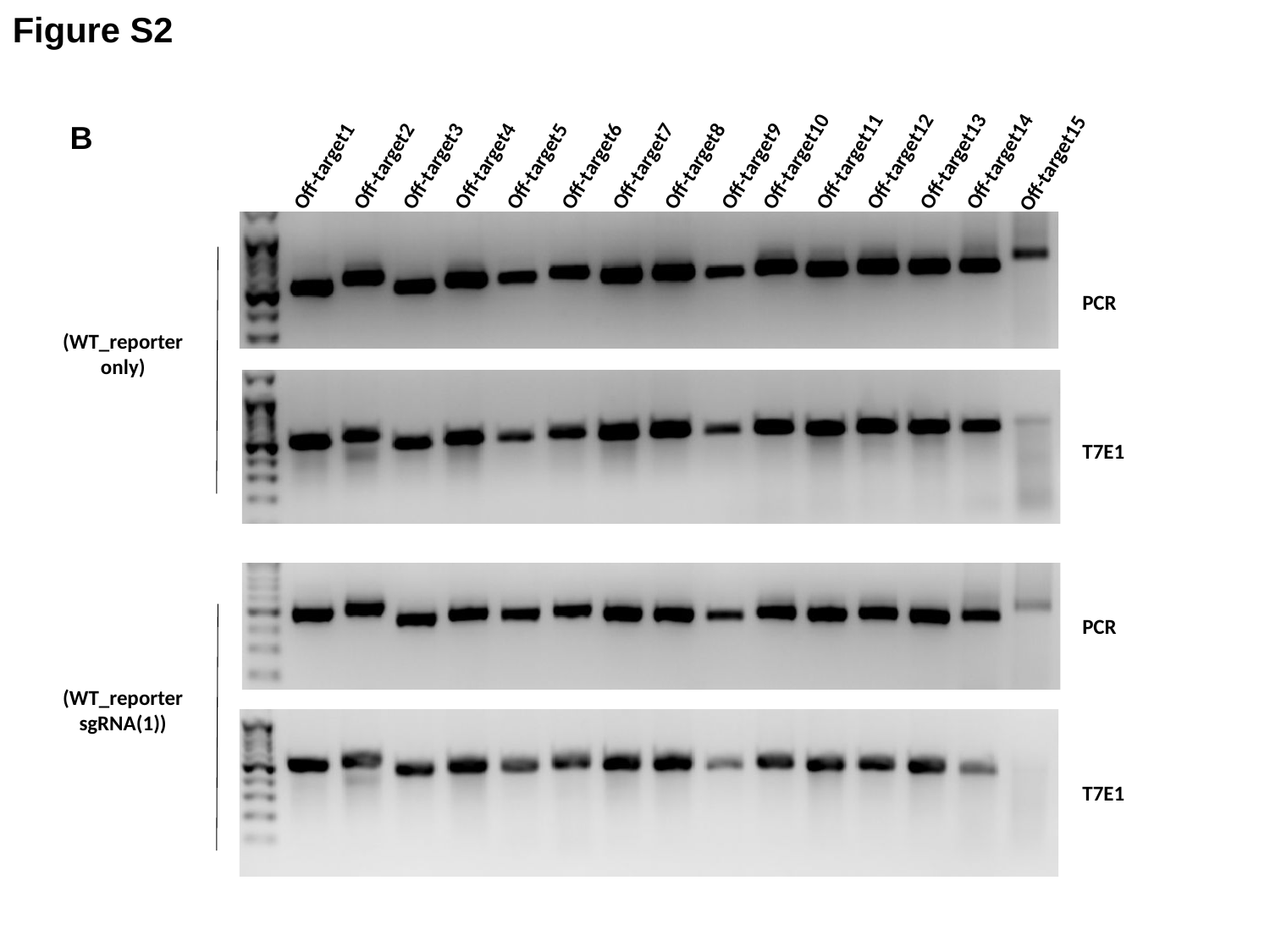

Figure S2
B
Off-target14
Off-target11
Off-target15
Off-target10
Off-target12
Off-target13
Off-target5
Off-target1
Off-target3
Off-target6
Off-target7
Off-target9
Off-target2
Off-target4
Off-target8
PCR
(WT_reporter only)
T7E1
PCR
(WT_reporter
sgRNA(1))
T7E1
